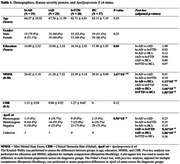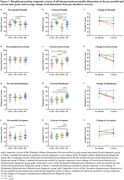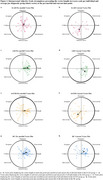# Pre‐morbid personality and personality changes in patients with the behavioral variant of Alzheimer's disease

**DOI:** 10.1002/alz70857_105756

**Published:** 2025-12-26

**Authors:** Fleur H.C. van der Linden, Kaitlin B Casaletto, Argentina Lario Lago, Faatimah Syed, Elsmarieke van de Giessen, Rik Ossenkoppele, Gil D. Rabinovici, Katherine P Rankin, David N. Soleimani‐Meigooni

**Affiliations:** ^1^ Memory and Aging Center, Weill Institute for Neurosciences, University of California San Francisco, San Francisco, CA, USA; ^2^ Memory and Aging Center, UCSF Weill Institute for Neurosciences, University of California, San Francisco, San Francisco, CA, USA; ^3^ University of California San Francisco, San Francisco, CA, USA; ^4^ Vrije Universiteit Amsterdam, Amsterdam UMC location VUmc, Amsterdam, Netherlands; ^5^ Alzheimer Center Amsterdam, Vrije Universiteit Amsterdam, Amsterdam UMC, Amsterdam, Netherlands

## Abstract

**Background:**

The behavioral variant of Alzheimer's disease (bvAD), an atypical Alzheimer's syndrome, clinically resembles the behavioral variant of frontotemporal dementia (bvFTD), but anatomically aligns more with typical amnestic AD (tAD). A distinct pre‐morbid personality predisposing bvAD patients to develop early behavioral deficits during neurodegeneration may explain this dissociation. However, personality (pre‐morbid and current) remains uncharacterized in bvAD.

**Method:**

In this retrospective case‐control study, 19 bvADs were identified and matched by age and sex to 25 tADs, 28 bvFTDs, and 37 healthy controls (HCs) from the University of California, San Francisco (UCSF) Memory and Aging Center (Table 1). Pre‐morbid and current personality were measured using the informant‐based interpersonal adjective scale (IAS). Composite‐t‐scores were calculated for each IAS dimension (warmth‐coldness, introversion‐extroversion, dominance‐submissiveness, and arrogance‐ingenuousness). Scores of all IAS traits were combined into a vector, which provided information about a person's overall personality disposition within the IAS circumplex. Between‐group differences were analyzed using ANOVA or Kruskal‐Wallis tests. In each group, differences between the two time points were assessed using paired t‐tests or Wilcoxon rank tests.

**Result:**

Pre‐morbidly, bvADs, bvFTDs, and HCs were colder than tADs (*p* = 0.004, 0.006, and 0.004, respectively), and bvADs and HCs were more ingenuous than tADs (both, *p* = 0.03) (Figure 1a, j). After symptom onset, bvADs and bvFTDs decreased in warmth (*p* = 0.003 and 0.0001), extroversion (*p* = 1.02*10^‐5^ and 6.86*10^‐6^), and dominance (*p* = 8.95*10^‐5^ and 8.88*10^‐6^) (Figure 1c, f, i). HCs and bvFTDs (*p* = 0.0001 and 0.01) increased in arrogance, while bvADs (*p* = 0.18) did not change significantly (Figure 1l). Additionally, bvADs and bvFTDs showed greater increases in vector length than tADs (*p* = 0.04 and 0.01) and HCs (*p* = 0.04 and 0.01), indicating a rigid personality that was dominated by coldness, introversion, and submissiveness (Figure 2).

**Conclusion:**

BvADs had a pre‐morbid personality comparable to bvFTDs and HCs, while tADs were characterized by higher warmth. After symptom onset, bvADs developed a rigid personality similar to bvFTD. These findings suggest that pre‐morbid behavior could be a relevant factor for understanding development of divergent behavioral phenotypes emerging in response to neurodegeneration. Moreover, significant personality changes are not exclusive to bvFTD.